# Get Healthy, Stay Healthy: Evaluation of the Maintenance of Lifestyle Changes Six Months After an Extended Contact Intervention

**DOI:** 10.2196/11070

**Published:** 2019-03-12

**Authors:** Brianna S Fjeldsoe, Ana D Goode, Philayrath Phongsavan, Adrian Bauman, Genevieve Maher, Elisabeth Winkler, Jennifer Job, Elizabeth G Eakin

**Affiliations:** 1 Cancer Prevention Research Centre School of Public Health The University of Queensland Brisbane Australia; 2 Prevention Research Collaboration Sydney School of Public Health The University of Sydney Sydney Australia

**Keywords:** maintenance, mHealth, physical activity, exercise, diet, overweight, body mass index, text messages

## Abstract

**Background:**

Extended intervention contact after an initial, intensive intervention is becoming accepted as best practice in behavioral weight control interventions. Whether extended contact mitigates weight regain in the longer term or it simply delays weight regain until after the extended intervention contact ceases is not clear.

**Objective:**

This study aimed to evaluate, in multiple ways, maintenance of weight, diet, and physical activity outcomes following Get Healthy, Stay Healthy (GHSH), a text message–delivered extended contact intervention.

**Methods:**

Clients completing the Get Healthy Service (GHS) lifestyle telephone coaching program were randomized to receive GHSH (n=114) or standard care (no additional contact, n=114) and were assessed at baseline (following completion of GHS), 6 months (following completion of GHSH), and 12 months (noncontact maintenance follow-up). At all 3 assessments, participants self-reported their body weight, waist circumference, physical activity (walking and moderate and vigorous sessions/week), and dietary behaviors (fruit and vegetable serves/day, cups of sweetened drinks per day, takeaway meals per week; fat, fiber, and total indices from the Fat and Fiber Behavior Questionnaire). Moderate-to-vigorous physical activity (MVPA) was also assessed via accelerometry. Maintenance was examined multiple ways: (1) using traditional methods to assess and compare group averages after some period of noncontact (ie, at 12 months), (2) using a novel approach to assess and compare group average changes over the first 6 months of noncontact, and (3) exploring individual participant changes (increase/decrease/no change) over the first 6 months of noncontact.

**Results:**

Retention over the 12-month trial was high (92.5%, 211/228). Participants had a mean (SD) age of 53.4 (SD 12.3) years and a baseline body mass index of 29.2 (SD 5.9) kg/m^2^. The between-group differences detected at 6 months were still present and statistically significant at 12 months for bodyweight (−1.33 kg [−2.61 to −0.05]) and accelerometer-assessed MVPA (24.9 min/week [5.8-44.0]). None of the other outcomes were significantly favored compared with the control group at 12 months. Changes over their first 6 months of noncontact for the GHSH group were significantly better than the control group in terms of accelerometer-measured MVPA and self-reported moderate activity (other differences between the groups were all nonsignificant). In addition to the maintenance seen in the group averages, most intervention participants had maintained their behavioral outcomes during the first 6 months of noncontact.

**Conclusions:**

The GHSH participants were better off relative to where they were initially, and relative to their counterparts, not receiving extended contact in terms of MVPA. However, based on the between-group difference in bodyweight over the first 6 months of noncontact, GHSH does appear to simply delay the *inevitable* weight regain. However, this delay in weight regain, coupled with sustained improvements in MVPA, has public health benefits.

**Trial Registration:**

Australian New Zealand Clinical Trials Registry ACTRN12613000949785; https://www.anzctr.org. au/Trial/Registration/TrialReview.aspx?id=364821&isReview=true

## Introduction

### Background

A large body of evidence on the maintenance of weight loss and/or behavior change following the end of initial interventions resoundingly shows a *relapse effect*, characterized by weight regain and/or behavioral decline back toward baseline levels [1-3]. This has led to a concerted focus on weight loss maintenance interventions, defined in the current literature as “extended contact” interventions—the intervention that continues after initial weight loss intervention, typically at a comparatively lower intensity than the initial intensive phase of intervention. Extended contact interventions have been consistently shown to somewhat mitigate the relapse effect [4-6]. What is not known is whether extended contact helps mitigate weight regain in the longer-term or whether it simply delays weight regain until after the extended intervention contact ceases.

Moreover, 2 previous studies evaluating text message–delivered, extended contact interventions for weight loss maintenance have shown that 6 months after extended contact ceases, participants’ body weight has remained, on average, significantly reduced compared with baseline [7,8]. However, another 2 studies that compared an extended contact intervention with a control condition after 2 months [9] or 6 months [10] of no contact found no between-group maintenance effects, although notably neither of these interventions achieved significant intervention effects at the end of text message–delivered extended contact. Research is needed to understand whether the improvements gained through extended contact behavioral interventions can be sustained following the end of such interventions, particularly in comparison with a control condition.

The “Get Healthy, Stay Healthy” (GHSH) intervention was an extended contact program delivered via text messages for 6 months following completion of an initial 6-month community-wide lifestyle telephone coaching program called “Get Healthy Service” (GHS) in Australia [11]. The GHSH intervention was evaluated in a randomized controlled trial (RCT) compared with normal practice following GHS (no ongoing intervention contact). Anthropometric (weight and waist circumference) and behavioral (physical activity and dietary) indicators were assessed at baseline (following completion of GHS), 6 months (following completion of GHSH), and 12 months (no-contact maintenance follow-up). We have previously reported that the GHSH intervention was feasible to deliver using semiautomated Web-based technology and was highly acceptable to participants [12]. Changes in body weight and physical activity (but not dietary outcomes) between baseline and 6 months were significantly better for the GHSH intervention group compared with the control group [12].

### Objectives

This paper aims to evaluate maintenance beyond the period of the GHSH extended contact intervention in multiple ways. First, we used the traditional method of assessing and comparing changes from baseline contemporaneously after some period of noncontact (ie, at 12 months, which is after 6 months of noncontact for the intervention group and after 12 months of noncontact for the control group). This method establishes whether an intervention has a lasting effect, after allowing some time for intervention recipients to relapse. Second, we used a novel approach to directly assess and compare the degree of changes over the first 6 months of noncontact between the intervention group (between 6 and 12 months) and the control group (between baseline and 6 months). This method adds to the previous by determining whether the degree of changes after extended-contact intervention are any different to changes that naturally occur over the same amount of noncontact time without extended contact. Finally, both of these maintenance perspectives consider only changes at the group level. We, therefore, also examined individual-level changes (personal increase/decrease/no change) over the first 6 months of noncontact, to explore to what extent nonsubstantial changes in group averages reflect all or most participants making no changes or are due to large increases by some participants being offset by large decreases by other participants.

## Methods

### Study Design

A detailed description of this RCT is published elsewhere [11]. Eligible consenting participants were randomized in a 1:1 ratio to the GHSH intervention and control groups, via a randomization website, by a research assistant with no involvement in participant recruitment. Randomization was across 2 strata (≥ or < the median of 3 kg weight loss during GHS). Recruitment began in August 2012 and 12-month follow-up data were collected until August 2014. Ethical clearance was received from the Human Research Ethics Committee at The University of Sydney (Protocol number: 03-2011/13523).

### Participant Recruitment

The GHS is available to adults (≥18 years and older) residing in New South Wales, Australia and is available for free via self-referral and health professional referral. Participants completing GHS between August 2012 and February 2013 were eligible to join the GHSH trial if they had no intention of re-enrolling in GHS coaching, were not involved in other GHS evaluation substudies, and owned a mobile phone. All eligible clients completing the initial contact intervention (GHS) within the recruitment time frame were invited to participate in GHSH during their final coaching call. Interested participants were mailed an information sheet and consent form and then contacted via telephone to establish their eligibility and willingness to participate. Verbal consent to participate was audio recorded, and participants returned a signed consent form via reply-paid post.

### The Extended Contact Intervention

The GHSH-extended contact intervention was delivered via individually tailored text messages. Tailoring data were collected during an initial and an interim telephone call (around 12 weeks), during which participants worked with a trained coach to set a 12-week weight goal (weight maintenance or further weight loss) and two 12-week goals for physical activity and/or dietary behavior change, with targets consistent with national guidelines [13,14]. For each behavioral goal (diet and/or physical activity), participants were asked to identify rewards for reaching their goal, expected benefits, preparatory behaviors for goal attainment, barriers and solutions, and a person who could support them to reach their goals. Participants selected their desired number of text messages (from 3-13 per fortnight), timing of texts (eg, 6 am), and type of texts. Overall, 4 types of texts targeted different behavior change strategies, each with different permitted frequencies: prompts to self-monitor weight (once per fortnight), goal checks for behavioral goals (from once per fortnight to once per week for each goal), real-time behavioral prompts (from none to 4 per fortnight for each goal), and goal resets for weight and behavioral goals (1 in week 6 and 1 in week 18). At 12 weeks, participants received a second telephone call from their coach to update their tailoring goals and preferences.

### Control Group Treatment

To minimize trial attrition, control participants were posted brief written feedback of results following each assessment. The control group received no other contact.

### Data Collection

Details of the data collection are reported elsewhere [11]. Briefly, data were collected at baseline, 6 months, and 12 months. Most outcomes were collected by computer-assisted telephone interviews (CATI), conducted by a research assistant, who was initially blinded to group allocation (information collected in the interviews limited this blinding at 6 and 12 months). The outcomes and measures were the same as those collected in the initial intervention, with the addition of an objective monitor of physical activity and a nutrition assessment tool, the Fat and Fiber Behavior Questionnaire (FFBQ) [15].

#### Anthropometric Outcomes

During the interviews, participants reported their body weight in kilograms (while wearing light clothes and no shoes) and waist circumference. Use of measurement aids during the interview was encouraged (scales and study-provided measuring tapes). Body mass index (BMI) was calculated based on self-reported height at GHS baseline and self-reported weight at each assessment point.

#### Physical Activity Outcomes

Self-reported physical activity included the number of weekly sessions spent: walking for 30 min or more, doing other moderate-intensity physical activity for 30 min or more (termed *moderate*), and doing vigorous-intensity physical activity for 20 min or more [16]. Further, objectively measured time spent engaged in moderate-to-vigorous physical activity (MVPA) was measured using the Actigraph GT1M—a dual-axis accelerometer. The protocol, published elsewhere [11], required participants to wear the accelerometer on the hip for 7 days during all waking hours. MVPA was assessed using a commonly implemented method [17] in which 60-second epochs with 1952 cpm or greater on the vertical axis were summed for each day of wear and averaged per wear day. Nonwear time, which was identified by an algorithm with published validity [18], was excluded along with nonwear days (<10 hours wear).

#### Dietary Behavior Outcomes

Dietary outcomes were recalled based on the participant’s usual behavior in the past month and included daily servings of fruit and of vegetables [19], average daily consumption of sweetened drinks, and takeaway meals per week [20]. Additional outcomes were the FFBQ’s 13-item fat index, 7-item fiber index, and 20-item total index, all of which were calculated as the average of the relevant items measured on a scale from 1 to 5 with higher values respectively indicating healthier habits concerning fat intake, fiber intake, or both.

### Sample Size

As previously reported [11], the sample size had been chosen a priori to provide 90% or more power to detect the following expected differences between groups in primary outcomes with 5% 2-tailed significance: 2 sessions per week of self-reported MVPA, 1 daily serving each of fruit and vegetables, 2 kg body weight, and a 4 cm waist circumference. The study was not powered a priori for questions concerning within-groups changes. For the FFBQ indices, fruit intake, takeaways, and sweetened drinks only, power was adequate (≥80%) to detect differences between groups meeting the minimum differences of interest (MDI). The MDIs were set at 1 kg weight, 1 cm waist circumference, 30 min or 0.5 sessions/week physical activity, 0.5 servings per day of fruit and vegetables, 0.5 takeaway meals per week, 0.25 cups per day of sweetened drinks, and 0.2 units on the FFBQ indices [12].

### Statistical Analysis

Maintenance is considered in three ways. First, whether anthropometric and behavioral outcomes are comparatively better after noncontact (at 12 months) for those who received the GHSH extended contact intervention than for those who had not (controls), second, comparing changes during the first 6 months of noncontact within the GHSH intervention group (ie, between 6 and 12 months) with changes in the control group (ie, between baseline and 6 months), and finally, considering behavioral maintenance at the individual-level during the first 6 months of noncontact in the intervention group.

Statistical analyses were performed using SPSS Statistics version 22 (IBM, USA) and STATA version 13 (StataCorp LP, USA). Significance was set at *P*<.05, 2-tailed. Changes within groups were assessed using paired *t* tests. All differences between groups were estimated adjusting for the same potential confounders as per the main outcome evaluation of the GHSH intervention [12]. Differences between the groups in their changes (for all outcomes and all time frames) were assessed using separate linear regression models adjusting for baseline values of the outcome and confounders. When assessing change over the period from baseline to 12 months, baseline values were taken as the beginning of the GHSH evaluation. When assessing changes over the first 6 months of noncontact, baseline values were taken as the beginning of the GHSH evaluation for controls or at 6 months for the intervention group. Group differences in daily values of accelerometer MVPA (log-transformed) were assessed using generalized estimating equation models, which accounted for repeated measures (1-7 days per participant per assessment), adjusted for confounders, and controlled comparisons for baseline values via the method outlined by Twisk [21]. Marginal means of the appropriate back-transformed expression were used to obtain the contrasts in minutes per week. The absence of substantial changes in group averages can be suggestive of maintenance; however, this can be achieved by large worsening in some participants being offset by others’ large improvements and does not necessarily show whether individuals maintained their personal outcomes. Accordingly, we further describe how many of the intervention participants maintained their outcomes during noncontact (ie, individual-level maintenance).

## Results

### Participants

Participants who remained in the study at 12 months had an average (mean [SD]) age of 53.4 (SD 12.3) years and baseline BMI of 29.2 (SD 5.9) kg/m^2^ (Table 1). Approximately two-thirds of the participants were female. Retention over the 12-month trial was high overall (92.5%, 211/228), but slightly lower in the intervention group (86.8%, 99/114) than in the control group (98.2%, 112/114; *P*=.002). Those lost to follow-up at 12 months (n=17) had significantly heavier baseline BMI, were more likely to smoke at baseline, and reported consuming fewer vegetables and more sweetened drinks at baseline than those who participated in the 12-month follow-up CATI (n=211; see Table 1).

### Sustained Improvement After Noncontact

**Figure 1:**
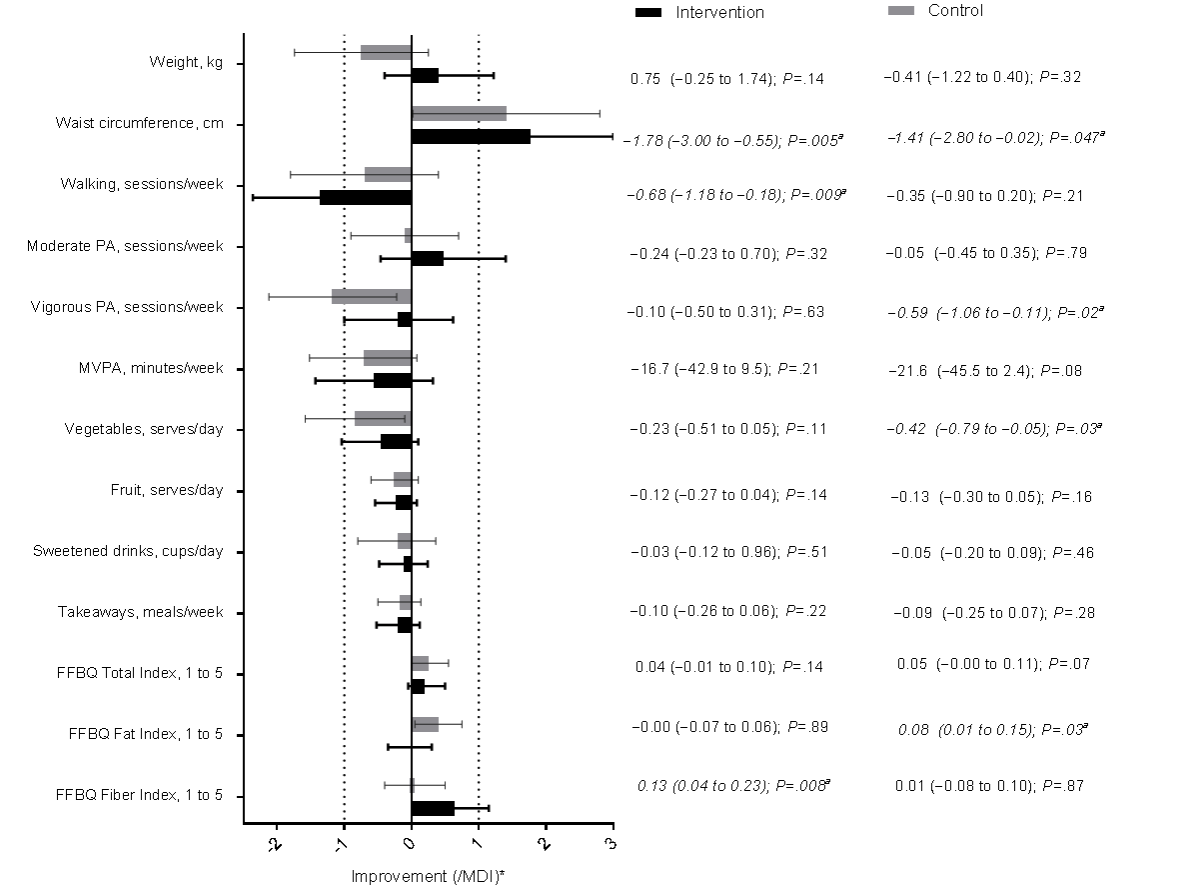
Mean changes (95% CI) between baseline and 12 months in study outcomes plotted as multiples of the minimum difference of interest (MDI) in the Get Healthy, Stay Healthy (GHSH) intervention (n=114) and control (n=114) groups ("a" indicates significant change *P*<.05. Asterisk indicates that the x-axis values for the means and CI are displayed as the mean, upper limit, and lower limit divided by the MDI value. Missing data are excluded for the intervention group or control group: n=13/3 [weight], n=14/6 [waist circumference], n=12/3 [self-reported physical activity and diet outcomes], and n=16/9 [accelerometer moderate-to-vigorous physical activity, MVPA]). FFBQ: Fat and Fiber Behavior Questionnaire; PA: physical activity.

Figure 1 shows changes from baseline to 12 months in study outcomes in the control group (12 months of noncontact after the initial GHS intervention) and in the GHSH intervention group (following 6 months of GHSH extended contact then 6 months of no contact). Results are described in units but plotted relative to a substantial decline or worsening (ie, MDI), to indicate whether the absence of a significant change was more consistent with maintenance (ie, a substantial worsening is unlikely) or an inconclusive result (ie, the error is too large to draw a conclusion). Over 12 months, the GHSH intervention group made significant improvements to waist circumference and the fiber index score, and the control group significantly improved their waist circumference and fat index score. The only significant declines between baseline and 12 months were self-reported walking, which declined in the intervention group, and self-reported vigorous activity, which declined in controls (Figure 1). Self-reported moderate physical activity and most of the dietary outcomes were maintained in both groups.

Although both groups had displayed a large degree of behavioral maintenance at 12 months, extended contact was still associated with a significant advantage over control treatment for body weight *(P=*.04) and accelerometer-assessed MVPA (*P*=.01), with differences between groups averaging approximately 1.3 kg and 25 min per week (Table 2). Only small and nonsignificant differences between groups were seen with the other outcomes; however, CI included meaningful differences in self-report physical activity and vegetable intake.

### Changes During the Noncontact Period After Extended Care

**Figure 2:**
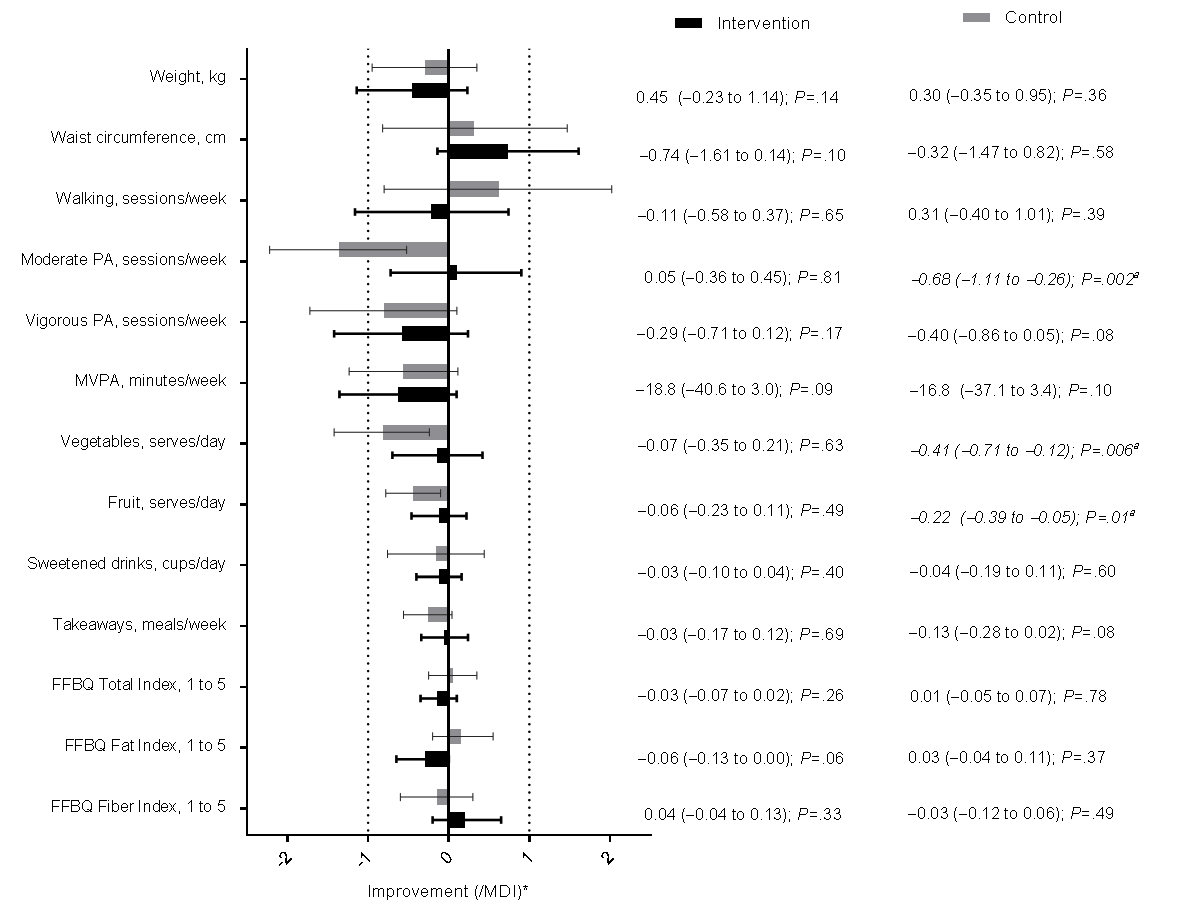
Mean changes (95% CI) over first 6 months of noncontact in study outcomes plotted as multiples of the minimum differences of interest (MDI) in the Get Healthy, Stay Healthy (GHSH) intervention (n=114) and control (n=114) groups ("a" indicates significant change, *P*<.05. Asterisk indicates that the x-axis values for the means and CI are displayed as the mean, upper limit, and lower limit by the MDI value). FFBQ: Fat and Fiber Behavior Questionnaire; PA: physical activity.

Figure 2 shows the changes over the first 6 months of noncontact within each group. Statistically significant changes over the first 6 months of noncontact occurred only in the control group, not in the intervention group following extended contact. All of these changes were worsening of outcomes rather than improvements (Figure 2). Intervention changes (all nonsignificant) during this time frame were suggestive of maintenance for waist circumference, moderate activity, and all of the dietary outcomes, but margins of error precluded definitive conclusions concerning the other outcomes.

When compared with the control group changes over their first 6 months of noncontact, those receiving intervention fared significantly better than those receiving usual care in terms of accelerometer-measured MVPA and self-reported moderate activity (Table 2). Other differences between the groups were all nonsignificant. All were small (except for walking) and a substantial effect of extended contact was unlikely for weight, FFBQ indices, takeaways, sweetened drinks, and fruit intake (based on the CI). However, CI included potentially meaningful differences in waist circumference, walking, vigorous physical activity, and vegetable intake.

### Individual-Level Maintenance of Outcomes During Noncontact

**Figure 3:**
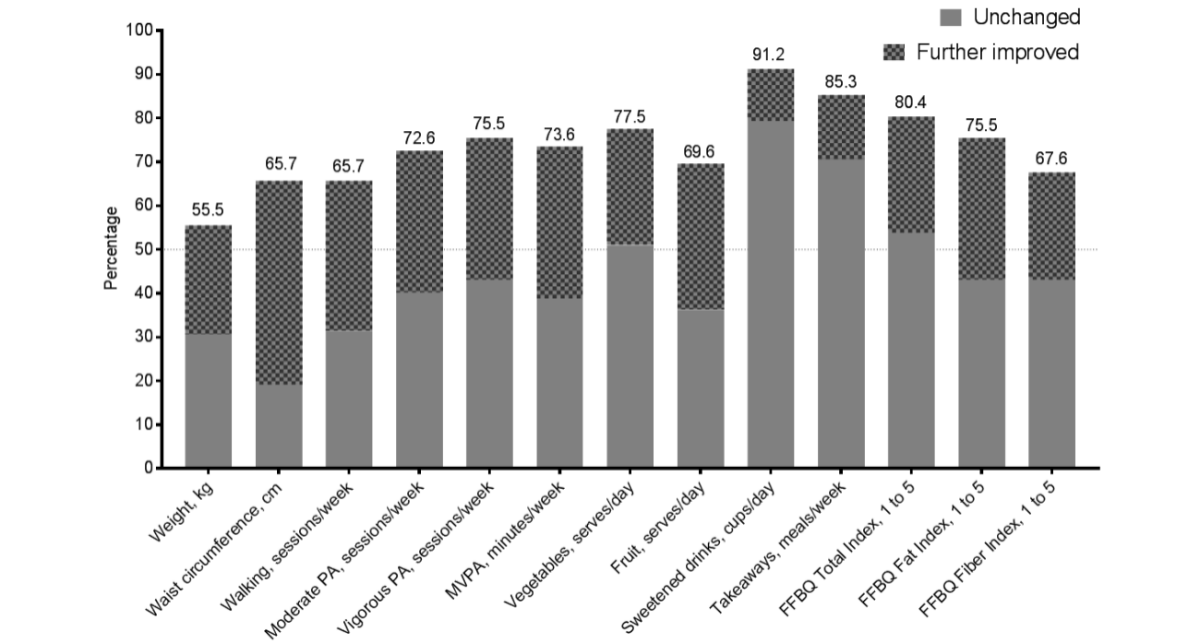
Percentage of the Get Healthy, Stay Healthy (GHSH) intervention participants (n=114) maintaining (by either no change or further improvement) their study outcomes during the first 6 months of noncontact. FFBQ: Fat and Fiber Behavior Questionnaire; MDI: minimum difference of interest MVPA: accelerometer moderate-to-vigorous physical activity; PA: physical activity.

In addition to the maintenance seen in the group averages, many intervention participants had maintained their behavioral outcomes during 6 months of noncontact, whereas a minority of participants had worsened in their outcomes during that period. Figure 3 shows the percentage of intervention participants who had maintained their outcomes during noncontact. Nearly all outcomes were maintained (ie, no worsening of ≥ the MDI) by the majority of participants. The proportion of maintenance was lowest for weight (55.5% [56/101]) and highest for sweetened drinks (91.2% [93/102]), with a substantial proportion of maintenance coming in the form of further improvement for most outcomes. Notably, weight regain was still reasonably common (44.5% [45/101]), despite this maintenance intervention and despite the overall intervention effects for weight outcomes.

**Table 1:** Baseline characteristics of participants by study group and for those who remained in the study at 12 months and those who dropped out by 12 months.

Characteristics		GHSH^a^ intervention (n=114)^b^	Control (n=114)^b^	Retained at 12 months (n=211)^c^	Lost to follow-up (n=17)^c^	*P* value^d^
**Health and demographics**						
	Age (years), mean (SD)	55.5 (12.3)	51.2 (11.9)	53.4 (12.3)	52.9 (12.6)	.87
	Body mass index (kg/m^2^), mean (SD)	29.3 (5.8)	29.6 (6.3)	*29.2 (5.9)^e^*	*32.6 (7.2)*	*.03*
	Weight (kg), mean (SD)	82.8 (19.4)	83.6 (18.9)	82.6 (19.3)	89.9 (16.2)	.13
	Waist circumference (cm), mean (SD)	98.9 (15.4)	99.6 (14.9)	99.0 (15.2)	103.1 (14.3)	.29
	Gender (female), n (%)	74 (64.9)	78 (68.4)	140 (66.4)	12 (71)	.48
	Paid employment (response: yes), n (%)	69 (61.1)	68 (59.6)	129 (61.1)	8 (50)	.43
	Education (postschool qualification), n (%)	73 (64.0)	77 (67.5)	136 (64.5)	14 (82)	.19
	English at home, n (%)	109 (96.5)	111 (97.4)	205 (97.2)	15 (94)	.41
	Indigenous Australian, n (%)	1 (0.9)	5 (4.4)	6 (2.9)	0 (0)	—^f^
	SEIFA^g^ (percentage in most advantaged 3 quintiles), n (%)	86 (75.4)	78 (68.4)	151 (71.6)	13 (77)	.79
	Region (percentage in major cities), n (%)	71 (62.3)	82 (71.9)	144 (68.2)	9 (53)	.28
	Initial health (percentage ≤ “fair”), n (%)	25 (21.9)	30 (26.3)	49 (23.2)	6 (35)	.25
	Current smoker, n (%)	5 (4.4)	7 (6.1)	*9 (4.3)*	*3 (18)*	*.05*
**Physical activity (PA)**						
	Accelerometer PA (minutes/week), mean (SD)	196.9 (144.4)	196.2 (143.6)	195.1 (136.2)	214.4 (221.3)	.60
	Vigorous PA (sessions/week), mean (SD)	1.56 (1.86)	2.33 (2.53)	1.9 (2.3)	2.1 (2.2)	.75
	Moderate PA (sessions/week), mean (SD)	1.11 (1.78)	1.60 (1.97)	1.4 (1.9)	0.8 (1.4)	.13
	Walking PA(sessions/week), mean (SD)	3.99 (3.04)	3.30 (2.44)	3.6 (2.7)	4.8 (3.7)	.20
**Dietary behaviors**						
	Vegetable (servings/day), mean (SD)	3.1 (1.4)	3.4 (1.8)	*3.3 (1.7)*	*2.6 (0.8)*	*.005*
	Fruit (servings/day), mean (SD)	2.0 (0.9)	2.0 (1.0)	2.0 (1.0)	2.0 (1.0)	.95
	Sweetened drinks (cups/day), mean (SD)	0.2 (0.5)	0.4 (0.9)	*0.3 (0.8)*	*0.1 (0.3)*	*.04*
	Takeaways (meals/week), mean (SD)	0.5 (0.8)	0.5 (0.9)	0.5 (0.8)	0.8 (1.3)	.38
	FFBQ^h^ total score (1-5), mean (SD)	3.3 (0.4)	3.3 (0.4)	3.3 (0.4)	3.2 (0.3)	.54
	FFBQ fat score (1-5), mean (SD)	3.5 (0.5)	3.5 (0.5)	3.5 (0.5)	3.5 (0.4)	.89
	FFBQ fiber score (1-5), mean (SD)	2.9 (0.5)	2.9 (0.5)	2.9 (0.5)	2.8 (0.4)	.44

^a^GHSH: Get Healthy, Stay Healthy.

^b^Figures exclude missing data; that is, 1 GHSH intervention participant (employment, English spoken at home, referral source, and accelerometer moderate-to-vigorous physical activity) and 1 control participant (waist circumference and indigenous status).

^c^Figures exclude missing data: n=1 lost to follow-up (employment, English at home, and waist circumference).

^d^*P* value for difference between those retained and those lost to follow-up determined by independent samples *t* test (continuous variables) or chi-square test (categories).

^e^A statistically significant difference between those lost to follow-up at 12 months (n=17) and those who participated in the 12-month follow-up computer-assisted telephone interview (n=211).

^f^Invalid chi-square test (not presented).

^g^Socioeconomic indices for areas (SEIFA), specifically the Index of Relative Socioeconomic Advantage and Disadvantage (IRSAD).

^h^FFBQ: Fat and Fiber Behavior Questionnaire.

**Table 2:** Difference in study outcomes between the Get Healthy, Stay Healthy (GHSH) extended contact group (n=114) and control group (n=114) adjusted for baseline values of the outcome and potential confounders^a^.

Outcome		Baseline to 12 months^b^		First 6 months of noncontact^c^	
		Mean difference (95% CI)	*P* value	Mean difference (95% CI)	*P* value
**Anthropometry**					
	Weight (kg)	− *1.33 (−2.61 to −0.05)*^d^	*.04*	0.01 (−0.94 to 0.95)	.99
	Waist circumference (cm)	−0.60 (−2.33 to 1.12)^e^	.49	−0.72 (−2.13 to 0.69)^e^	.31
**Physical activity (PA)**					
	Accelerometer PA, minutes/week	*24.9 (5.8 to 44.0)*	*.01*	*18.3 (0.8 to 35.7)*	*.04*
	Walking PA, sessions/week	−0.07 (−0.69 to 0.55)^e^	.83	−0.51 (−1.34 to 0.32)^e^	.23
	Moderate PA, sessions/week	−0.11 (−0.62 to 0.39)^e^	.66	*0.53 (0.07 to 0.99)*	*.03*
	Vigorous PA, sessions/week	−0.12 (−0.64 to 0.40)^e^	.66	−0.32 (−0.84 to 0.21)^e^	.23
**Dietary behaviors**					
	Vegetables, serves/day	0.10 (−0.32 to 0.53)^e^	.63	0.17 (−0.20 to 0.53)^e^	.36
	Fruit, serves/day	−0.00 (−0.22 to 0.21)	.98	0.10 (−0.11 to 0.32)	.35
	Sweetened drinks, cups/day	−0.06 (−0.19 to 0.06)	.32	−0.07 (−0.21 to 0.08)	.36
	Takeaways, meals/week	−0.10 (−0.26 to 0.06)	.22	−0.02 (−0.18 to 0.13)	.77
	FFBQ^f^ total index, 1 to 5	−0.02 (−0.09 to 0.06)	.64	−0.02 (−0.10 to 0.05)	.51
	FFBQ fat index, 1 to 5	−0.08 (−0.17 to 0.01)	.08	−0.08 (−0.17 to 0.01)	.08
	FFBQ fiber index, 1 to 5	0.09 (−0.03 to 0.20)^d,e^	.13	0.08 (−0.04 to 0.19)	.19

^a^Mean differences (intervention−control) adjusting for confounders as per the main GHSH evaluation and baseline values of the outcome as estimated using linear regression, or generalized estimating equations for repeated measures for accelerometer data (1-7 days per assessment per participant).

^b^Missing data are excluded for intervention group/control group: n=13/3 (weight), n=14/6 (waist circumference), n=12/3 (self-reported physical activity and diet outcomes), and n=16/9 (accelerometer physical activity).

^c^With baseline values of the outcome taken as values at the beginning of the noncontact period (GHSH baseline in the usual care group and at 6 months upon cessation of extended care in the intervention group). Missing data were excluded for the intervention group/control group: n=13/2 (weight), n=15/2 (waist circumference), n=12/2 (self-reported physical activity and diet), and n=19/6 (accelerometer physical activity).

^d^Significant difference between control and intervention group favoring intervention.

^e^Inconclusive: nonsignificant comparison but meaningful differences contained within the 95% CI.

^f^FFBQ: Fat and Fiber Behavior Questionnaire.

## Discussion

GHSH is an extended contact intervention offered after completion of a free, publicly available lifestyle telephone coaching program. Our evaluation [12] previously found that at the end of GHSH, both weight and accelerometer-measured MVPA were significantly better in the intervention group than in the control group, and the present evaluation showed that these between-group differences were still present and statistically significant at 12 months. None of the other outcomes, such as self-reported physical activity and dietary behaviors, were significantly favored compared with the control group at 12 months.

The presence of intervention effects following a noncontact period are commonly interpreted as indicators of maintenance of the intervention’s effectiveness [22,23], but in isolation of other evidence (eg, the direction of change in individuals), this interpretation can be problematic. Therefore, in this study, we also considered the lack of substantial regression toward baseline levels as indicating outcomes were maintained. In GHSH, only self-reported walking declined significantly over 12 months in the group that received extended contact, with most other outcomes maintained or being further improved. Furthermore, during the noncontact period following extended contact (6-12 months), the intervention group did not significantly worsen in any outcomes, instead improving or maintaining outcomes. Over both of these time frames, there was some uncertainty around the maintenance of self-reported vigorous physical activity and vegetable intake, with wide margins of error failing to rule out worsening in these outcomes as being unlikely in general as opposed to just not observed in our sample.

These findings clearly indicate, relative to their counterparts not receiving extended contact, and relative to their initial levels, participants receiving extended care were “ahead of the game.” Although useful, this perspective of maintenance fails to consider how extended care generates such gains. *Does it seem to mitigate the rate of behavioral decline upon withdrawal of intervention contact, or merely postpone behavioral decline (until after extended contact)?* Using a novel approach, we tested this question by comparing both groups in their changes over the same amount of noncontact time (6 months), beginning immediately after the intervention ceases (GHS or GHSH that involved extended care). This approach ensures that not just the amount of time (6 months) is consistent in comparing groups but also the timing relative to withdrawal of intervention contact. The changes over this key time frame significantly favored the intervention group in terms of accelerometer-measured MVPA and self-reported moderate activity, suggesting extended care helped to reduce the relapse effect for this behavior. However, there was no substantial or significant difference in body weight between groups over the first 6 months of noncontact. So, to some extent, the extended contact intervention promoted *maintenance* merely by delaying weight regain in the intervention group. This delay in weight regain meant that there was an extended period of continued or maintained weight loss, which, although not directly measured in this trial, should have public health benefits for participants’ physical health [24]. Importantly, this extended period of continued or maintained weight loss is very promising, given that GHSH is a low resource, text message–delivered public health program offered free to participants. Furthermore, the extended contact did help participants to maintain their physical activity behaviors, which independent of changes in weight, should also bring public health benefit [25].

Individual-level maintenance is also important, but seldom considered [1], as average changes at the group level can be stable without individuals necessarily maintaining their personal changes. In GHSH, during the first 6 months of noncontact (whether following extended or initial contact), most of the outcomes were maintained by most individuals, with at least a two-thirds majority maintaining or further improving their outcomes over this time. The outcome for which most individuals had displayed a failure to maintain outcomes during noncontact (ie, worsening ≥MDI) was body weight, with just under half of the participants (intervention and controls) increasing weight by at least 1 kg.

Much has been written about the diversity of definitions applied to *maintenance* of behavior change [26-28]. Maintenance has been defined as being achieved when a significant intervention effect at the end of an intervention has been maintained after varying periods of no contact, which largely refers to the *success* of the intervention on group averages. Some authors have viewed maintenance as a criterion or threshold of amount of behavior change to be retained. Relatively few publications examine maintenance as individual trajectories. The findings of this evaluation, which examined maintenance from multiple perspectives, indicate researchers should similarly consider reporting maintenance of behavior change following interventions in multiple ways that help better understand how maintenance might occur, not just whether or not it occurred by some particular criterion for maintenance.

Evidence suggests need for ongoing support that people can access as required over long periods of time. Mobile technologies facilitate this type of ongoing monitoring and contact in cost-effective ways. There is international consensus that obesity is a chronic, relapsing disease process that requires continuous treatment [29]. Although it is important to point out that the participants in this study were not necessarily obese at baseline, the majority were working toward weight loss goals, and it is the mechanism of weight loss regain that needs ongoing treatment. Researchers and practitioners need to acknowledge that individuals will cycle in and out of multiple programs across a life span, and therefore, we should not be imagining a single intervention effect to be *maintained*. What we need to do instead is ensure that individuals have a positive experience in these programs so that they approach the next program with positive expectations and high self-efficacy.

This trial has tested the addition of tailored text messages to a telephone coaching program to extend the duration of care provided. It led to better outcomes for those receiving the texts, while the contact was maintained. As we move forward, we need to consider cost-effective mediums to maintain contact with people as they cycle in and out of weight loss and lifestyle support programs, and text messaging may be a feasible and affordable way to do this. Limitations of this trial include the reliance on self-reported anthropometric outcomes (albeit validated and reliable tools were used) and that the trial was underpowered to detect within-group changes. The strengths of this trial include comprehensively examining maintenance in 3 different ways, conducting an RCT within partnership and in a service delivery context, and inclusion of high-quality behavioral measurement tools. These strengths and the positive maintained outcomes of the GHSH intervention have resulted in this evaluation directly informing the addition of an extended contact program in GHS. More pragmatic research trials such as, this one, need to be conducted to generate practice-based evidence to inform service delivery decisions. Finally, future evaluations of the maintenance of intervention impacts should consider reporting these in multiple ways, including a comparison of group averages when holding the period of noncontact equal and individual patterns of change.

## Abbreviations

BMI: body mass index
CATI: computer-assisted telephone interview
FFBQ: Fat and Fiber Behavior Questionnaire
GHS: Get Healthy Service
GHSH: Get Healthy, Stay Healthy
MDI: minimum differences of interest
MVPA: moderate-to-vigorous physical activity
RCT: randomized controlled trial

## Appendix

Multimedia Appendix 1 CONSORT‐EHEALTH checklist (V 1.6.1).
